# RETRACTION: Intervention Mechanism of Hunag‐Lian Jie‐Du Decoction on Canonical Wnt/β‐Catenin Signaling Pathway in Psoriasis Mouse Model

**DOI:** 10.1155/ecam/9756925

**Published:** 2026-07-23

**Authors:** Evidence-Based Complementary and Alternative Medicine

RETRACTION: X. Yang, G. Luo, L. Fu, et al., “Intervention Mechanism of Hunag‐Lian Jie‐Du Decoction on Canonical Wnt/β‐Catenin Signaling Pathway in Psoriasis Mouse Model,” *Evidence-Based Complementary and Alternative Medicine*, 2022, 3193572, https://doi.org/10.1155/2022/3193572.

The above article, published online on 14 April 2022 in Wiley Online Library (https://wileyonlinelibrary.com), has been retracted by agreement between the authors and John Wiley & Sons Ltd. The retraction has been agreed due to concerns reported on PubPeer [1]. Specifically:

• In Figure 2a, there appears to be unexpected, similar regions between the ß‐catenin/IMQ + HLJDD (0.475 g/mL) panel and the c‐Myc/IMQ + HLJDD (0.950 g/mL) panel
• In Figure 4a, the Cyclin D1 and TBX3 bands appear highly similar

The authors state that these errors were introduced due to confusion in figure preparation, and have requested to retract the article in light of these concerns.
